# Panel and Patient

**Published:** 1923-06

**Authors:** 


### PANEL AND PATIENT.

### "THIS PICTURE AND THAT."

"THE experiment of trying to provide 15,000,000 people with medical attendance on contract lines has had ten years' trial, and has proved a failure."

"PENNY-IN-THE-SLOT" DIAGNOSIS.

This statement was made by Dr. Edwin Smith' the Coroner for North-East London when criticising the panel system at a Shoreditch inquest on a man whose father complained of the treatment his son had received:—

"It is common knowledge that at a typical panel consultation the patient announces what seems to him to be his most prominent symptom—a cough or a pain, or so forth —and instantly he gets a prescription for something supposed to fit his condition. There is no time for any but the most perfunctory investigation, and he is hurried from the surgery almost before he has entered it. The underlying cause of the symptoms must be missed in an immense number of cases, and a vast amount of disease overlooked. The human machine does not lend itself to this lightning diagnosis and penny-in-the-slot style of treatment. The panel system was a retrograde step, not too strongly described as a disastrous blunder and a miserable failure. It put a premium on scamped work and inefficiency, for it could not be denied that, on the whole, the worst work was being done by the men drawing the largest incomes. Criticism of a purely destructive kind is unsatisfactory, and if asked for suggestions I would say, replace the present system, after consultation and agreement, with a body truly representative of the doctors, by one under which the doctor is paid for the actual work done and the patient is free from restrictions both in choosing and in changing his medical attendant."

DR. SMITH'S "NONSENSE."

In reply, an official statement, signed by Dr. Alfred Cox, the Medical Secretary, has been issued by the British Medical Association. Dr. Cox writes:—

"So far from there being 'widespread and increasing dissatisfaction of the general public,' with the panel system, all the evidence the British Medical Association has is to the effect that the system has greatly improved during the past two or three years, that the evidences of dissatisfaction are largely based on a few individual bad cases, and that the representatives of insured persons are of opinion that with the goodwill of the medical profession (which they will have) the system, which it must be remembered has been a great national experiment, can easily be made into one with which all concerned can feel satisfied. Dr. Smith's statement that there is no time for any but the most perfunctory examination by the panel doctor is nonsense. The panel doctor is simply a general practitioner who happens to be doing part of his work under the panel system, and if a panel patient requires a thorough examination the doctor can give it to him just as well as to a private patient—and he generally does. Dr. Smith is wrong when he suggests that the panel doctor necessarily has a very large number of cases to deal with. The average panel doctor has less than one thousand insured persons on his list; no panel doctor in any area is allowed to have more than 3,000, and in many districts the highest possible limit is 2,500 or 2,000."

Dr. Cox adds that the first of Dr. Smith's suggestions for the improvement of the service has been considered by the Approved Society Consultative Council and "turned down," and that the second has the support of the medical profession.

### Burned Hospitals.

On the average two hospitals and two asylums are burned down in the United States every week.

